# On the Sickness and Mortality in the French Army, during the Campaign in Turkey and the Crimea in 1854-56

**Published:** 1858-07

**Authors:** Gavin Milroy

**Affiliations:** Fellow of the Royal College of Physicians, and Member of the Sanitary Commission to the British Army in the East.


TRANSACTIONS

OF THE

EPIDEMIOLOGICAL SOCIETY OF LONDON.

### Papers and Communications.

### ON THE SICKNESS AND MORTALITY IN THE FRENCH ARMY, DURING THE CAMPAIGN IN TURKEY AND THE CRIMEA, IN 1854-56.

By GAVIN MILROY, M.D., Fellow of the Royal College of Physicians, and Member of the Sanitary Commission to the British Army in the East.

MILITARY medicine presents a singularly favourable field in many respects for the study of various epidemic diseases.

Their development and rise with the circumstances and phenomena which may precede or accompany it, their course and progress, and their decline and disappearance, can all be watched among a large body of men with the utmost exactitude. Then, the several conditions which favour their occurrence, or which modify their type, are under the immediate observation of the medical officer. He can trace and follow each case from its very commencement, and may thus more easily and accurately discover the cause or causes which appear to have produced it. The constitutions and former health of his patients are known to him. He can ascertain where they have been, how they have been occupied, whether they have been exposed to fatigue, or to peculiar atmospheric vicissitudes, and whether they have been in communication directly or indirectly with other persons affected with the disease. Their diet and clothing, the quality of their food and drink, as well as the nature and condition of their accommodation, are under his continual cognisance.

It is a point of the greatest importance in all epidemiological inquiries to determine beyond dispute, and to record at the time, the exact dates of the first few cases of attack in an epidemic outbreak, with the circumstances attending the occurrence of each case; for it is upon the accurate knowledge of these particulars that some puzzling questions respecting the origin and the mode of spreading of the disease can alone be satisfactorily solved. In the army, this information may always be had. And if the medical officer will take the trouble to obtain at the same time like information respecting the disease among the civil population in the immediate neighbourhood of his barrack or cantonment, and also among the ships of war on the station, if he be placed at or near a seaport, his testimony would go far to settle several of the most disputed problems of ætiological medicine.

The influence, too, of different climates, and of different topographical sites, as to elevation, geological features, etc. on the same disease, can also be studied with peculiar advantage by the military physician.

The late campaign in the East afforded a good opportunity for the observation of certain epidemic diseases on a great and extended scale, and fortunately the opportunity has not been lost to medical science. Several valuable works illustrative of the subject have been published both in this country and in France. Of these, the *Relation Médico-Chirurgicale de la Campagne d'Orient*, by M. Scrive, who was at the head of the medical department of the French army throughout nearly the whole period, is especially worthy of notice and commendation. His detailed and connected record of the health of the army, from first to last, in this in every respect memorable campaign, is most instructive.

I have thought that a brief summary of the leading facts of his narrative, with a few comments and remarks *en passant* on the great truths which these facts ought to teach us, would form a not unsuitable subject for a paper to be read before a Society whose professed object is the investigation of the causes of diseases, with special reference to their mitigation and prevention. Almost all the details will be taken from M. Scrive's work; a few only from other sources of information.

The first French troops, about 8000 in number, landed at Gallipoli, on the European shore of the Dardanelles, at the end of March, 1854. Like all other Turkish towns, Gallipoli is very filthy and unwholesome; narrow winding streets, with stinking black gutters in the middle, and heaps of foul rubbish everywhere on the surface. Most of the houses in the lower part of the town are huddled together, dark and unventilated; the amount of abomination underneath them can only be guessed at. The well water is often impure. The climate of Gallipoli is fine and considered to be healthy; it is not unlike that of the south of France and north of Algeria.

The troops were camped out a few miles beyond the walls. Two or three buildings in the town were taken possession of and converted into a hospital, but without due preliminary precautions having been adopted to fit them for that purpose. The result was that they had very soon to be abandoned, in consequence of their unwholesomeness. This was invariably found to be the case throughout the whole campaign, whenever native buildings were made use of for the sick, without a previous thorough purification below as well as above ground, and the existing means of ventilation being much improved.

By the beginning of May, 24,000 men had arrived at Gallipoli. There was very little sickness among them; and the only ailments were angina and bronchitis, with a few cases of intermittent fever, chiefly among the soldiers who had come from Algeria. In another month, the force had become nearly doubled. The sick-rate still continued low, not exceeding 15 per thousand of the strength. Among the fever cases at this time were a few of the typhoid type. The other diseases were the same as in May. No epidemic sickness existed. The men were fairly fed, and the camp was on favourable ground.

In consequence of the threatened fall of Silistria on the Danube, most of the troops were moved forward during June from the Dardanelles into Bulgaria, in order to be near the seat of war. By the beginning of July, the bulk of the army was concentrated around Varna, situated on the shores of the Black Sea. They were camped chiefly on the wooded heights above the town. A large Turkish barrack within the walls was prematurely occupied for a hospital; and again, as at Gallipoli, the step proved an unwise one; the building had ere long to be evacuated. For not only did the sick not recover favourably in it, but many cases of spontaneous fever occurred among the patients admitted for other ailments—*an infallible proof of unwholesomeness from some cause or other within.*

At this period, the strength of the French army in the

East had been raised to about 55,000 men. With the increasing heats of summer, the sick-list had risen rapidly, and the character of the prevailing maladies had undergone a change. The ratio of sickness was now between 30 and 40 per thousand of the strength, and was caused chiefly by bowel disorders, along with a good many cases of intermittent and bilious remittent fevers, the latter being not unfrequently associated with symptoms of a typhoid character. Many of the bowel attacks—both among the recent arrivals and among the troops which had first landed—assumed a decided choleraic type; and already sporadic solitary cases of malignant cholera (*cholera foudroyant*) had occurred at different points of the French occupation in Turkey. In the last week of June, a rapidly-fatal case happened at Varna in a zouave who had been two months in the country; and in the following week, a second case, which proved fatal in the course of a few hours. As, moreover, the *cholerine* cases had very much increased both in number and in severity at this time, M. Scrive began to dread a serious outbreak of the pestilence among the troops in and around Varna. He accordingly took the wise precaution of recommending that the tents in the encampment should be occasionally shifted from one spot to another—that they should be spread out more apart from each other—that the number of men in each tent should be reduced by one half; also that the men should be required to wear their abdominal belts at all times; that they should not be exposed to be wetted or chilled, especially at night; that the camping grounds should be thoroughly cleansed and disinfected; and that a most strict surveillance should be kept over the men, to check all attacks of diarrhœa from the very first. The food and drink, too, of the troops were also modified and regulated, as far as circumstances would permit, and their fatigue duties were abridged as much as possible.

These prudent measures would doubtless have had a most salutary result, had not circumstances, as we shall presently see, occurred to prevent their execution by calling off the great bulk of the troops elsewhere.

Simultaneously with the manifestation of cholera at Varna, evidences of its agency made their appearance among the French troops not only at Constantinople and Gallipoli, but also at the Piræus in Greece. The Russian army on the Danube also was suffering at this time from the pestilence.

Several of the transports, which left Marseilles and Toulon towards the end of June and in the beginning of July, had cases of cholerine and cholera during their voyage out, and were obliged to land some of their sick at Malta and at Gallipoli.

The French transports, it may be remarked, were generally so much crowded that very many of the men had to remain on deck, night and day, without shelter or sufficient covering. The state of their between-decks was, of course, always most impure. Besides cholera, typhoid fever was not unfrequent on board many of them. A good many cases of small-pox occurred in several transports.

I would here observe that, independently of the amount of actual disease among troops during a voyage, it is to be borne in mind that men landing from a crowded unwholesome ship are much more liable to sickness from endemic or epidemic disease in a new country, than others who have been better accommodated, and have had the advantage of good food and shelter, with pure air, throughout the voyage. The mischief of neglect on this head is therefore twofold—prospective as well as present. This is a point in military hygiene which has not attracted the full attention which it deserves.

Throughout July, cholera was on the increase among the French troops at every station, with the exception of Adrianople, where it did not appear till the second week of August. The general sick-list of the army had nearly doubled during the first three weeks of the month, and every arrival of new regiments from France or Algeria swelled the number.

M. Scrive having observed that almost all the bad cases of cholera at Varna had occurred either among the inmates of the hospital in the town, or among the troops quartered within the walls, and that the troops camped outside the town were comparatively exempt from the disease in its malignant form, determined to empty the hospital as much as possible; ordered no more patients to be sent into it; and had all the cases in future treated in tents pitched out on the plateau beyond the walls, separating the tents well from each other, and at some distance from those of the troops, and keeping them all thoroughly ventilated. The good effects of these measures were soon obvious, and made, he tells us, a very strong impression upon his mind. These rules, he justly remarks, should invariably be acted on in the repressive and preventive treatment not of cholera alone, but of all pestilential diseases without exception.

It was at this period, while the choleraic influence prevailed extensively among the troops, that the disastrous movement into the Dobrudscha—the notoriously malarial region on the south side of the Danube, near its embouchure —was made, with the view of threatening the left flank of the Russian army, and also in the hope, as the military authorities supposed, of exercising a favourable diversion on the *morale* of the men, who had become, as usual, depressed by inaction and increasing sickliness. *The medical staff were not consulted upon the occasion.*

Although no particulars are made known, it is pretty obvious that the needful precautionary measures for the health of troops in such an expedition, and at that season of the year when malarial diseases are always most rife and active, were entirely neglected by the officers in command.

On the 20th of July, the first division, forming the advanced column, moved from Varna. After the first day's march, several men were attacked with cholera. Four or five days then passed over without any fresh cases of a malignant character; but on the 26th—the men having now become much fatigued by successive forced marches further and further into the marshy region, and subjected all the while to most trying privations—a terrible explosion of the pestilence took place. It was like that which occurred among a British force at Kurrachee, near the mouth of the Indus, twelve years ago, only on a larger and more destructive scale. Hundreds of men were struck down at once and died within a few hours after being seized. From day to day, things became worse and more appalling. All attempts to abate the storm were in vain. The medical officers were left without any resources. Within a short week, between two and three thousand men of the division had been smitten, and more than two-thirds of the cases had quickly proved fatal. In one regiment alone, advancing inland from Kostendjie, 300 of the men were attacked within twenty-four hours, and most of them died on the spot. Appalled by the blow, the commanding officer sought to retrace his steps at once; but before the regiment could reach the coast, more than half its strength had perished, and large numbers reached it only to expire miserably on the beach.

Upon its return to Varna, says M. Scrive, the first Division, which was 12,000 strong on leaving France, did not muster more than between seven and eight thousand bayonets. Among the victims were two general officers and nine medical officers.

The second Division, which had advanced much less far into the Dobrudscha, and had consequently encountered far less fatigue and privations, suffered proportionally less; while the third Division, forming the rear of the expedition and having advanced but a little way from Varna, was comparatively unscathed.

The history of this expedition affords a very striking proof of the influence of excessive fatigue (coupled doubtless with unsuitable and unwholesome food and drink) in a malarial district, in giving deadly force to the cholera poison. Medical experience in India and other tropical countries can furnish us with many similar instances.

While the expeditionary army in Bulgaria was suffering so dreadfully from the pestilence, the troops stationed in and around Constantinople and Gallipoli were all, more or less severely, affected by it. At the same time too, the regiments which had been sent to the Piræus suffered a large amount of sickness and mortality. The evil effects of taking possession of old buildings, without due preliminary sanitary preparations, were experienced there as everywhere else.

It is estimated that the entire loss sustained by the French army in the East from cholera alone, during the months of July and August, amounted to between seven and eight thousand; upwards of five thousand having died on the spot, and the rest disabled by protracted illness, if indeed a large proportion of them did not subsequently sink under fever and other secondary diseases.

One pestilence had thus, in two short months, out of an army 55,000 strong, and before a shot had been fired, deprived France of as many men as were slain by the enemy in the field during the twelve months from the day of landing in the Crimea to the end of the siege by the capture of Sebastopol, and when the average strength of the army was nearly double the above number!

But besides cholera in July and August, other diseases, especially dysentery, typhoid and remittent fevers, had served to swell the sick-list. The total admissions into hospital in these two months amounted to nearly 10,000—or between a fifth and a sixth part of the whole force under arms.

That much of this sickness might have been avoided, had the men, both the sick and the well, been less crowded together, often too in unwholesome localities, and had more attention been paid from the first to the selection of places for hospitals and encampments, and if also the quality of the supplies—the food, drink and clothing—had been better and more suited to the wants of the troops and the circumstances of their varying condition, is distinctly asserted by all the French medical officers.

Before we pass on to a new field of observation and experience—from Turkey to the Crimea—the highly interesting and important question as to the probable origin or source of development of the pestilence, which had caused such havoc in the army, presents itself for our consideration. Did it spring up in the places where it appeared? or was it brought to and imported into these places, as has been asserted, from France or Algeria by the troops which were continually arriving from these countries, Turkey itself being previously free and intact? In a social as well as a military point of view, and as affecting the welfare of nations and communities not less than it may affect the movements of troops, and the general operations of war, the question is obviously one which demands special attention from our profession.

M. Scrive and the other medical officers present with the French army in Turkey when the cholera first appeared among the troops, and who could trace its progress step by step, are of opinion that the disease developed itself on the spot, and was not primarily introduced from without. Besides a general and increasing prevalence of *cholerine,* sporadic cases of the disease of the most malignant type had occurred both at Varna and on the Dardanelles before the arrival of any infected troops; and, indeed, the first manifestations of the cholera that year, 1854, were observed quite as early in Turkey as in France; viz., in the latter half of June. Towards the close of the previous year, too, it had existed in Bulgaria and other parts of the Turkish dominions, as well as the southern provinces of Russia, the Russian army in Bessarabia having suffered severely from it in the autumn of 1853.

These facts seem to show that the manifestation of the pestilence in the summer of 1854 was not dependent on the arrival of infected troops from France (as has been stated in some documents published in this country), but rather that it developed itself in its own mysterious way in the places where it appeared; although, doubtless, the continual influx of infected and highly predisposed troops served to aggravate the evil and greatly to increase the mortality, especially when sanitary precautions in the lodging and feeding of such troops were overlooked or neglected. I shall afterwards have occasion to revert to the influence of climate, season, and local conditions on the spread or otherwise, etc., of cholera as it was observed in the allied army in the East. The scene now changes.

It was on the 7th of September, that the allied fleets sailed from Balshik on the Bulgarian coast; on the 13th they anchored off Eupatoria. The next three days were spent in landing the troops. After halting for a couple of days, the combined armies advanced on the 19th, and next day fought the battle of the Alma. The French army, 23,000 strong on that occasion, had 300 killed and 900 wounded.

A few isolated cases of cholera had occurred during the voyage to Eupatoria; but, on the whole, the health of the troops was considered to be good on landing in the Crimea. The number of attacks of cholera increased very considerably immediately after the battle, in consequence chiefly, it was believed, of the armies halting, for two days, on the field where hundreds of putrid carcases of horses were scattered about, and the surface of which was moreover polluted with ordure from its occupation by the Russians. Other causes, however, had a share. The troops had undergone very great fatigue for several days under a hot sun, and with insufficient food, which moreover was not of first-rate quality. It deserves notice, as bearing on this subject, that at this time the officers suffered quite as much from cholera as the men; the rations of both were the same, and the former had, of course, no means of adding to or improving them. Throughout the campaign generally, however, the proportion of officers attacked by cholera was much less than that of the men;—their food and their accommodation were better.

In military and naval epidemiology, this point—the relative numbers of attacks and deaths among officers and men—should always be ascertained and stated. It is often highly suggestive.

During the march to the south side of Sebastopol, bowel complaints became more numerous, and a good many deaths from cholera took place. Among other victims, Marshal Arnaud was attacked at this time, and died a few days afterwards on shipboard. He had long suffered from disease of the heart. The want of water on the march was a source of intolerable distress to the poor sufferers.

In the month of October, the first of the memorable siege, the hospital admissions from disease alone (exclusive of gunshot wounds) amounted to not less than between a seventh and eighth part of the whole force, then about 46,000 in number. Nearly a fourth of the admissions were from cholera, and more than two-thirds of the deaths were caused by it. The newly-arrived troops suffered most. This was invariably the case throughout the whole campaign. It is an important epidemiological fact in respect of cholera, as of yellow fever and other like diseases, that fresh comers into a locality or district where the pestilence exists, or has even just begun to manifest its existence, are much more liable to seizure than the residents. Hence doubtless, on many occasions, has arisen the idea of such persons having introduced a disease, merely from the circumstance of their being among the earliest victims.

The necessity for extra vigilance in the hygienic treatment of newly-arrived troops in a foreign climate is naturally suggested by this consideration.

Already traces of scurvy had become evident in the French army; it was often associated with diarrhœa and typhoid fever.

The rations were insufficient in quantity, and consisting, day after day, of the same salted indigestible meat, served to irritate the bowels, while they failed to nourish the system. "This was a serious state of things," says M. Scrive, "at a moment when the army had more than need of all its strength." Fortunately, the weather had hitherto been favourable, and the climate of the Crimea was anything but insalubrious.

In November the cholera subsided, but scorbutic diarrhœa and dysentery increased both in amount and in severity; and the death-rate to cases rapidly went up. Hitherto, wounds and operations had on the whole healed pretty favourably, but now they refused to unite. The total admissions, from wounds as well as diseases, this month amounted to a tenth part of the force in the field. Gunshot wounds occasioned only a fifth part of these admissions, although the sanguinary battle of Inkerman took place on the 5th (where the French had 300 killed and 600 wounded), and the siege works were being pushed on with great activity.

After the terrible storm on the 14th, the weather became more wintry, and the result was that a good many cases of frost-bite occurred in the latter half of the month. The men had only their miserable *tentes d'abri* for shelter, and their clothing was moreover quite insufficient for the season. The only two hospital huts in the army having been smashed by the storm, the whole of the sick and wounded were consequently in tents until some large hovels or *cubones* could be excavated in the ground and roofed over with the *débris* of the old huts and with earth, in order to receive the worst cases. These partially subterranean hovels cannot, of course, ever be made or kept wholesome for hospital purposes. They afford the means of shelter against inclement weather;—that is all that can be said for them. The atmosphere within is necessarily impure.

In December, things were much the same as in November, only worse. Besides numerous cases of scurvy and frost-bite, a good deal of typhus had sprung up in the camp. Cholera, too, continued to appear occasionally among the new arrivals. The first wooden huts for hospitals sent from France were received this month. They were insufficiently ventilated, and not provided with flooring,—a defect which unfortunately could not be supplied on the spot from want of materials.

The new year opened with sharper cold and ruder weather; but the men on duty were still without proper tents or suitable clothing, or even sufficient and right food to enable them to resist it. Not a few poor fellows were frozen to death. Between two and three thousand cases of severe frost-bite were received into the ambulances: in nearly half the cases, the loss of some member or extremity ensued. No fewer than 800 of these sufferers died, either in the camp, or subsequently at Constantinople.

The hospital admissions this month were higher than they had yet been. Nine thousand fresh cases were sent into the ambulances, and upwards of six thousand sick and wounded were shipped off to Constantinople—an aggregate of fully fifteen thousand sick out of a nominal force of 78,000 in the field. Vast numbers, moreover, who could not be received into hospital, were unfit for duty.

But the miseries of the troops had not yet reached their acme. February was still more disastrous in sufferings than January—sufferings arising from privations and neglect, and which might, therefore, have been in a great measure or altogether prevented. Scorbutic disease prevailed everywhere. Three thousand men were sent into hospital with it; but these were only the worst cases, for the whole army was at this time more or less deeply tainted. And no wonder. Fresh vegetables had never been served out, and the wild *taraxacum,* which the men had been told to gather for themselves to make a salad, was now not to be had! Neither lemon-juice nor vinegar seems to have been supplied to the French troops at any time throughout the campaign.

Typhus had increased considerably this month in the camp, and it was spreading in the hospitals to other patients and to the attendants. Several of the medical officers died of it. As may be anticipated, wounds at this time would not heal, and a good many cases of hospital gangrene occurred. "Nous sommes fort mals à tous les points de vue" are the expressive words of the principal medical officer in his report at this dismal period.

The more favourable weather in March allowed a general cleansing and purification of the camp to be begun. The tents, and more especially the hospital ones, were pitched on fresh sites, and they were directed not to be dug or hollowed out as they had been during the winter, and also to be more thoroughly ventilated. Chloride of lime was to be occasionally sprinkled on the earth floors. The heaps of filth and rubbish were burned, or, when that could not be done, a strong solution of sulphate of iron was spread upon them;—the same application was used to deodorise the latrines. Carcases of horses, etc. were buried a couple of feet below the surface, and covered with a layer of quick lime. Charcoal is not mentioned among the disinfectants used. The medical officers urged the paramount necessity of fresh meat and vegetables being provided for the troops; and meanwhile, the men were recommended to recommence getting all the *taraxacum* they could find—a poor substitute, it must be confessed, for regular supplies of potatoes or onions.

In April, these various sanitary measures were carried out more efficiently, and the health of the troops certainly improved. Still, there was a large amount of scurvy and also of typhus fever in the camp. Both these formidable maladies, however, were now sensibly on the decline. Cholera, which had all but ceased in March, increased somewhat in April. It was not till the beginning of the following month that the increase was considerable; but then the progress of the disease steadily and rapidly advanced. Large reinforcements were continually arriving at this time, and it was among these, as usual, that most of the cases occurred. The Imperial Guard, which had been stationed for some weeks at Constantinople, and where they were suffering severely at the time from the pestilence, were among the arrivals in May. Although they were still affected with it on leaving the Bosphorus, and although the disease existed also around Sebastopol when they landed at Kamiesch, the change to the Crimea proved beneficial. The cholera abated, and the general health of the men improved, while the strength of the besieging army was greatly enhanced by these choice troops. Care was taken that their tents were pitched well apart from each other, kept thoroughly ventilated, and not crowded.

Great praise, it seems to me, is due to M. Scrive for the judgment he showed in the whole business. He had been consulted by General Canrobert as to the propriety of moving up the Imperial Guard from Constantinople, while they were still under the influence of cholera. On a due consideration of all the circumstances, M. Scrive thought that they might be so advantageously, provided proper precautions were taken. The result proved the wisdom of his advice, and may be fairly quoted as one out of many instances of the value of enlightened medical opinion in the operations of a campaign.

The irregularly erratic—one might almost say, the capricious—course and movements of the cholera in the camp might find an analogy in those of a blight in the vegetable world, or in the wanderings to and fro of insect swarms over a district, but were quite inexplicable on any hypothesis of contagious transmission, or of a generally and uniformly-diffused atmospheric contamination. The disease did not appear simultaneously in the two great divisions of the French army forming the left and the right attacks, but rather in succession in the one after the other. The second *corps d'armée,* operating against the Mamelon and Malakoff, suffered first; and it was not till a week or two later that the other *corps d'armée* at the extreme left began to be affected. One division of this latter corps, which formed part of the expedition to Kertch at this period, and which apppeared to be free from the disease when it sailed from Kamiesch on the 22nd of May, was attacked a few days after landing at Kertch, and therefore about the very same time as their comrades before Sebastopol.

About the end of this month, the sick list was swelled by a good many cases of intermittent fever, occurring chiefly among the troops which had taken possession of the marshy flat of the Tchernaya (recently evacuated by the Russians), or among the soldiers who had had the disease at Rome or in Algeria. Remittent fever, also, became more rife with the increased heats of summer; nor was typhus quite extinct in the hospitals, for more than one medical officer fell a victim to it in May.

June proved to be a very sickly, as well as a very sanguinary, month. Out of a force estimated at 122,000 men, little short of a sixth part was sent into hospital by disease and by gunshot wounds. The admissions from the casualties of war, compared with those from zymotic disease, were in the proportion of one from the former to between three or four from the latter; although the fierce actions on the 6th, when the Mamelon was taken, and on the 18th, when the unsuccessful attack was made upon the Malakoff, took place this month.

Cholera, which reached its acme in June, occasioned about a third part of the whole sickness; choleraic diarrhœa, dysentery, and malarial fever made up the rest. The weather was at times extremely hot, and there were occasionally heavy rains. As the siege-works were being pushed forward with great energy, the fatigue work of the trenches told, of course, also upon the health of the men.

These circumstances may explain, in part at least, the large amount of sickness from the diseases just enumerated, but will not prepare us for the re-appearance—or rather the increase—of scurvy at this season in the French army. The supply of fresh vegetables had as yet never exceeded a very scanty and irregular allowance; and now the summer heat, we are told, had withered up all the dandelion which for the last two or three months the poor fellows had been able to collect for themselves!

July brought an abatement of cholera; but malarial fevers and dysentery were very prevalent. Scurvy, too, was on the increase. Twelve hundred men were sent by it into the ambulances—as many as by cholera. But the mere number of admissions into hospital affords no idea of the extent of the former disease. "All our old soldiers, those who had been out since the beginning of the year, are more or less scorbutic," writes M. Scrive at this date.

In August, the number of fresh cases of scurvy taken into hospital was double that in July. Cholera had much declined; but bowel-complaints were still extremely rife. These, along with malarial fevers, caused the great bulk of the sickness. Altogether, the health of the French army was at this time anything but satisfactory. Between ten and eleven thousand sick and wounded were shipped off to Constantinople in the course of the month, and nearly sixteen thousand fresh admissions into the camp ambulances took place. In the battle of the Tchernaya, on the 16th, the French had 300 killed, and 800 wounded.

We come now to the crowning event of the campaign in a military point of view:—would that we could say, in a medical point of view likewise. But, alas! there is often no parallelism or synchronism between the successes and disasters of war in these two aspects.

On the 8th of September, Sebastopol fell by the glorious atchievement of the capture of the Malakoff by the French troops. Their wounded that day amounted to between 4000 and 5000. More than 500 wounded Russians were also received into their hospitals.

As there were fully 5000 sick and wounded in the ambulances at the time, hospital accommodation for upwards of 10,000 patients was at once required. Unfortunately, this did not exist; and the result was, that the huts and tents were excessively crowded; and hospital gangrene—the typhus of wounds—which had for months occurred only in isolated cases in the camp, speedily became frequent in every ambulance. Scurvy, too, continued to add a large quota to the sick list; and cholera had not ceased to attack, although not to a great extent, the young recruits soon after landing.

On the whole, however, there was less fresh sickness in September than in August, owing doubtless, in a great measure, to the heats of summer having given way to more cool and agreeable weather. The favourable change continued throughout October, and disease still further declined. Fresh cases of scurvy, although much less numerous than in the preceding month, were still far from being infrequent; and cholera still lingered in the camp, but with diminished power.

As from this period cholera ceased to play an important part in the sickness of the French army, it will be convenient here to state the general conclusions which M. Scrive formed respecting its character as it was seen in the Crimea. Since the day of the landing at Eupatoria, it had never been entirely absent from the French army; and yet at no time, not even in May or June, could it be said to prevail as an absolute epidemic in the camp. It certainly never acquired the force of a pestilence, as it did in the Dobrudscha; and it was always possible, by the adoption of sanitary precautions, to attenuate and mitigate its malignancy, and greatly to limit its extension. On all occasions its chief stress fell upon new comers: there was no exception to this law — for so it may be called—among the successive arrivals of the 250,000 French troops which at one time or another landed in the Crimea.

Notwithstanding the almost constant arriving of infected ships and men at Kamiesch, the disease never spread among the resident population of that place; and we have seen that, on various occasions, troops which landed with the disease among them seemed to get rid of it soon after they were spread about in their encampments in the front.

The experience of the British medical officers coincided with that of their French brethren. At Balaklava (and also at Malta), as at Kamiesch, the disease at no time spread on shore, although transports and other vessels were continually coming in with cases on board, and although many of the ships that were lying nearest to the foul margin of the harbour did not escape.

In the British camp, too, the existence of the cholera in one regiment or body of men never seemed to be the cause, or operating agency, by itself of its development among another regiment encamped near it. But the favouring influences of recent arrival, local insalubrities, and dietetic and regiminal irregularities were strikingly conspicuous on every occasion. M. Scrive's observations led him to a like result. It may be impossible, he truly remarks, to predict with any accuracy *where* or *when* the disease will fall or strike; but the circumstances which give force to the blow, and those which will mitigate it, are certainly known to us.

He considers that the emanations from the dejections of cholera patients, when many are treated together, may greatly favour the extension of the disease in hospitals, unless the most thorough ventilation be kept up.

The average duration of the outbreaks of cholera, among the French troops in the East, varied from two or three to five or six weeks. An invasion usually took from eight to fifteen days to reach its maximum force; and about the same time was spent in its decline. The disease never terminated abruptly, or all at once; but it kept lingering about for some time, often unexpectedly making itself felt in places which it had hitherto unaccountably spared; at other times, re-appearing in spots which it had already visited, and which it was believed that it had left. This feature of seeming capriciousness in its movements is not peculiar to cholera. Other epidemic diseases often exhibit the same character.

The tendency to secondary fever (which very generally proved fatal) was always observed to be greater during the decline, than during the rise and increase, of an epidemic invasion. In the Crimea, most of the fatal cases of cholera terminated in this way.

Rather more than six thousand deaths occurred from cholera among the French troops in the Crimea from September 1854 to December 1855. The number of cases had been between twelve and thirteen thousand. The proportion of attacks to the strength of the army in the field was greatest in October and November of the first year, and in June and

July of the second. It was lowest in March and December of 1855.

The recent arrival of the troops in the autumn of 1854, and the arrival of immense reinforcements in the latter part of the spring of 1855, in connection with the increased heat on the approach of the summer, had much to do with the greater prevalence of the disease on both occasions.

But to return to the course of our narrative. The commencing rude weather, in November 1855, found the French army almost as unprepared for encountering another winter on the bleak heights of the Crimea as it had been in the previous year, and its aggregate number was now nearly twice as great. The medical staff were, therefore, full of apprehensions at the prospect, knowing as they well did the cachectic condition of the troops generally, and the utter insufficiency of the means for the proper shelter and care of the sick and wounded. Large numbers of these poor fellows were still lying on the ground under canvas, often without any means of artificial warmth; nor was there the hope of better protection throughout the whole winter. If such was the condition of the invalids, it need scarcely be said that that of the men on duty was not better. They were huddled together in tents which did not exclude the rain, or in mud hovels deeply imbedded in the ground for the sake of better shelter, and kept close and unventilated to exclude the cold air. Besides this prolific source of unwholesomeness, the earth floors of these wretched abodes soon became polluted from various causes. All the fuel for cooking had to be cut down and brought from a distance; and as the extent of the line of defence which our allies undertook to occupy was very great, extending nearly from Baidar to Kamiesh, the strength of the French soldiers was then far more taxed than that of the British troops, while their condition in every respect as to shelter, food, and clothing, was infinitely worse. No one who witnessed the state of the French camps that winter can forget their thorough wretchedness everywhere. To me it is one of the most painful reminiscences of the field, only surpassed by the simultaneous horrors of the hospitals at Constantinople.

M. Baudens, who had been sent out from France to examine and report to the Emperor the state and the requirements of the army at this time, found, he says, the constitution of most of the old campaigners "used up" (*usure complet*), while that of the young recruits was in general extremely feeble; and then there was, he adds, no longer the excitement of the siege to "galvanize the courage of the soldiers."

With the greater inclemency of the weather in December, the number of the sick, as well as the gravity of their ailments, much increased. Thirteen thousand fresh cases of disease were sent into the hospitals in the camp during the month, and between four and five thousand invalids were sent down to Constantinople. Besides numerous bronchial and pulmonary attacks, disorders of the bowels had advanced both in frequency and severity, and formed the predominant cause of sickness in the army. The cold of winter seemed to aggravate this class of diseases as much as the heats of summer.

Scurvy, too, was on the increase, as was to be expected from the cold damp weather and the insufficient supply of fresh vegetable food. And now another terrible enemy began to raise its head. Typhus, which in the winter of 1854 had occurred but to a very limited extent, was much more prevalent than it had yet been in the camp; and it was, moreover, evidently spreading both in the regimental tents and cabanes among the men on duty, and also among the inmates and attendants of the ambulances. No one could wonder at this; every medical man foresaw the storm. The enemy was the very same that used to spring up in all the old dens of our prisons, and has ever proved the worst scourge of foul and crowded hospitals, and which, therefore, received the very appropriate appellation of "*jail*" or "*hospital*" fever.

The self-multiplying and propagating property of the engendered venom adds terribly to its power. So it proved in the French army. Each victim poisoned others near him; and soon it became impossible to determine what cases were of spontaneous development, and what were the results of contagious transmission. Curative medicine was, of course, at an end; nor could almost anything be done in the way of even mitigating the disaster, in the destitute condition of the troops in mid-winter. There were no means of segregating or dispersing the sick, nor of finding better accommodation for any of the troops, whether sick or well; and the hospital difficulties were still further increased by the fewer opportunities of sending off the sick to Constantinople,—at first, in consequence of the inclement weather, and at a later period, from the hospitals there becoming from mismanagement pest-houses of the worst description.

From week to week things rapidly became worse and worse. In January, there was double the number of new cases of disease that occurred in December; and February surpassed its predecessor in suffering and death. Seven-and-twenty thousand fresh sick were crammed into the ambulances during these two months. The increase both of typhus and of scurvy had become, says M. Scrive, truly awful; "it was the most terrible disaster of the whole campaign." Many of the medical officers fell victims to the fever, against whose ravages among their patients they could do nothing but look on in despair.

It is estimated that, from December 1855 to the following March, between nineteen and twenty thousand cases of typhus occurred among the French troops in the Crimea, and that nearly one half the attacked died on the spot. Among the victims were thirty-one of the medical officers, of whom seventy-five were attacked. During this period, too, upwards of 28,000 sick from other diseases were (we cannot say treated, but) sent into hospital, and of this number, about 7000, or a fourth part, perished. Moreover, between 27,000 and 28,000 had in the same interval been sent off to the hospitals at Constantinople. Of these, many thousands died either on board the transports, or soon after their arrival.

It is unnecessary to follow the medical history of the French army in the Crimea for the few remaining months after the conclusion of peace at the end of March, 1856. By the shifting of the different camping grounds, the emptying and purification of the foul abodes of the well and the sick, and the greater dispersion and better accommodation which could then be procured, as well as by the increased supplies of fresh food, the amount of disease and death rapidly subsided.

It was, indeed, high time; for it is believed, that the army could not muster for effective duty more than one-half its nominal strength—a state of things nearly as bad as that of the British force sixteen months before, when the terrible hardships of the trenches were superadded to the disastrous results of insufficient clothing, food, and shelter. How different now, when not above six or seven per cent. of the British army were off duty, and slight bronchial affections formed a principal part of the sickness. The Sardinian army was very much better conditioned and cared for than the French, and suffered proportionately less from disease this winter. Their state, however, was far from being equal to that of our countrymen. Typhus and scurvy continued to exist to a considerable extent. Their damp ill-ventilated dab-and-wattle huts, partially imbedded in the ground, however neat and snug to the eye, could not be so wholesome as was desirable; and their hospital huts, although most substantially constructed and well provided, were not half so well aerated as those in the British camp.

But it was not in the Crimea alone that the pestilence of typhus committed such dreadful ravages among the French during the winter of 1855-6. In consequence of the large drafts of sick and disabled continually being sent down from the camp to Constantinople, the hospitals there had become crowded to overflowing; and this, too, without the adoption of anything like the needful hygienic precautions. Not only was the ventilation of these buildings most imperfect, but their atmosphere was still further polluted with foul emanations from both within and without. The consequence was, that they very soon became breeding-places as well as reception-houses of disease. Typhus rapidly multiplied itself by the double process of spontaneous development and infectious multiplication. Vast numbers of the sick admitted with other maladies, as well as of the wounded, caught the fever and died; and few of the attendants, nurses, and orderlies escaped. Many of the medical officers perished.

In the first three months of 1856, upwards of 53,000 patients were (as appears from an official return given by Dr. Bryce in his able and instructive work, *England and France before Sebastopol*) treated in the French hospitals in and around Constantinople. Considerably more than a third part of the number perished—a terrible death-rate, certainly. Typhus was the chief destroyer; its coadjutors being scurvy, dysentery, hospital gangrene, and frost-bites. What proportion of this great mortality was fairly attributable to the gravity of the cases when the sick were landed from the crowded transports and first received into the hospitals on shore, and what proportion may be ascribed to the baneful effects of the poisoned atmosphere of these buildings, and to the diseases engendered, aggravated and multiplied thereby, it is not possible, of course, to decide with any precision. But the experience of our own hospitals at Scutari, in which the death-rate to cases treated during the first three months of 1855 was about nearly the same as it was in the French hospitals at Constantinople during the first three months of 1856, and where it afterwards fell so rapidly upon the carrying out of the required sanitary improvements, warrants us in saying that the mortality from the latter cause far, very far, exceeded that from the former—probably two or three times at least.

In narrating the medical history of the army in the camp during the winter of 1855-6, M. Scrive expresses his grief and astonishment to his countrymen that, in the present day, their brave soldiers should continue to suffer such a disastrous amount of sickness and death after all the experience of former wars, which have invariably told the same dismal tale, viz., that three-fourths, at the very least, of the losses of an army in the field are, not from the fire or sword of the enemy, or from any other unavoidable casualties of active service, but from certain perfectly well-known diseases, which are all, more or less, under immediate control, and which may therefore be in a very great measure, if not altogether, avoided. Of these, typhus and scurvy are two of the most formidable, as well as the most easily preventable. They are the inevitable products of certain well-ascertained conditions, and they may be generated at will as surely as any salt or other compound may be formed by the chemist in his laboratory. And yet it was these very two evils which, two short years ago, brought the noble army of a mighty nation, at the close, too, of a glorious campaign, to almost the verge of destruction.

That the French medical staff in the camp deserved no share of the blame in this matter must be patent to all who will read the simple narrative of events as given by M. Scrive; their urgent and repeated warnings and remonstrances were without avail, and when the storm came, they were left without resources. Can the same be said, or the same excuse be alleged, for their brethren at the Constantinople hospitals, in reference to the frightful mortality which occurred in them, and which equalled, if it did not exceed, that in the camp with all its miseries and destitution? I fear not; for however hampered and shackled the medical officers of the French army appear to be, and however dependent they are made on other departments for the equipment and management of hospital establishments, one cannot but believe that much might have been done by those gentlemen to diminish, if not to correct, the enormous sanitary evils which produced such disastrous results. The worst of those evils unquestionably was the excessive crowding without previously securing a corresponding increase of ventilation—and of ventilation, too, so arranged, *that fresh, pure air should reach every inmate, and that the vitiated respired*

*air of all should be carried off continuously, by night and by day.* Unless this most essential of all requirements is invariably and unceasingly provided, nothing but mischief must ensue. Far better that the sick, and especially the sick from fever, should be scattered about in large courtyards, or upon any open clean and dry ground, and be put under canvas or any sort of shelter, however imperfect, and with the want of every comfort around them, than that they should ever be accumulated within a building where there is not an adequate amount of space for each inmate, or where a pure atmosphere cannot be maintained by the constant renewal of unpolluted air at all times in the four-and-twenty hours. Nothing will compensate for the want of this prime necessary, which, thanks to an ever-beneficent Providence, may always and everywhere be had, while with it the value of every other curative agency or influence will be tenfold enhanced.

The following is a brief summary of the number of admissions into hospital, and of the deaths from all causes in the French army during the twenty months from landing in the Crimea to the conclusion of hostilities. The figures, especially as regards the loss of life, are probably much below the truth; but they serve, on the whole, to bring out some highly interesting and instructive conclusions.

Out of a force which averaged, during the above time, nearly 104,000, upwards of 193,000 men were sent into hospital—or from nine to ten thousand a month. Between a fourth and a fifth part of the whole admissions arose from gunshot wounds and other accidents or mechanical injuries. The rest were caused by disease. Of the 193,000 sick and wounded, about 115,000 were sent down to the hospitals at Constantinople; so that rather more than 78,000 were treated in the camp ambulances.

The mortality in the field stood thus:—Exclusive of 7500 slain in action, 28,400 died in hospital, or considerably more than a third part of those treated. Of these 28,400 deaths, about 4000, or a seventh part of the whole, arose from gunshot wounds and accidents, leaving fully six-sevenths of the whole mortality in the ambulances as the result of disease. Many of the deaths, moreover, among the wounded were caused by diseases caught in hospital.

The deaths in the hospitals at Constantinople, during the same period, amounted to nearly 28,000; the mortality in them being, therefore, only a trifle under that in the field ambulances.

As the entire number of deaths resulting from gunshot wounds and other accidents during the whole period of the twenty months is set down at 8500, out of an aggregate of 43,250 cases, there must have been about 4500 deaths from this cause in the hospitals on the Bosphorus, or between a sixth and a seventh part of the whole mortality there; it being always kept in mind that a large proportion of the deaths among the wounded were due to fever and hospital gangrene.

After the details which have been given in the preceding narrative, it seems scarcely necessary to attempt to particularise the relative amounts of sickness and mortality caused by different special diseases among the French army in the Crimea, especially as in a very large proportion of the cases, different morbid states or elements were blended and associated together, springing as they did from the same or similar causes.

To the above aggregate number of deaths from disease and wounds in the field and in the hospitals at Constantinople, must be added those which occurred on board transports between Kamiesch and the Bosphorus, or between the Bosphorus and France. These are not given in the French returns hitherto published. They must have amounted to several thousands.

Finally, it may be mentioned that, besides the total loss of life from all causes, and during the entire period of the campaign in the East, estimated in the official returns at nearly 70,000 (but which there is every reason to believe was greater by 8000 or 10,000 at least); 65,000 men out of the 309,268 sent from France and Algeria to the seat of war, were invalided in consequence of disablement from wounds or the effects of disease.

Such, then, are some of the fruits of War, even to a victorious army, in the latter half of the nineteenth century!

